# Using the Transversal Admittance to Understand Organic Electrochemical Transistors

**DOI:** 10.1002/advs.202410393

**Published:** 2024-11-25

**Authors:** Juan Bisquert, Scott T. Keene

**Affiliations:** ^1^ Instituto de Tecnología Química (Universitat Politècnica de València‐Agencia Estatal Consejo Superior de Investigaciones Científicas) Av. dels Tarongers València 46022 Spain; ^2^ Department of Engineering Electrical Engineering Division University of Cambridge Cambridge CB3 0FA UK; ^3^ Cavendish Laboratory Department of Physics University of Cambridge Cambridge CB3 0HE UK; ^4^ Department of Materials Science and NanoEngineering Rice University Houston TX 77030 USA

**Keywords:** admittance, diffusion, impedance spectroscopy, ionic transistors, mobility

## Abstract

The transient behavior of organic electrochemical transistors (OECTs) is complex due to mixed ionic‐electronic properties that play a central role in bioelectronics and neuromorphic applications. Some works applied impedance spectroscopy in OECTs for understanding transport properties and the frequency‐dependent response of devices. The transversal admittance (drain current vs gate voltage) is used for sensing applications. However, a general theory of the transversal admittance, until now, has been incomplete. The derive a model that combines electronic motion along the channel and vertical ion diffusion by insertion from the electrolyte, depending on several features as the chemical capacitance, the diffusion coefficient of ions, and the electronic mobility. Based on transport and charge conservation equations, it is shown that the vertical impedance produces a standard result of diffusion in intercalation systems, while the transversal impedance contains the electronic parameters of hole accumulation and transport along the channel. The spectral shapes of drain and gate currents and the complex admittance spectra are established by reference to equivalent circuit models for the vertical and transversal impedances, that describe well the measurements of a PEDOT:PSS OECT. New insights are provided to the determination of mobility by the ratio between drain and gate currents.

## Graphical abstract

A powerful model is developed to understand the gate‐stimulated ac admittance measurement of organic electrochemical transistors (OECT). The vertical impedance (mainly ionic) and the transversal impedance are described, and how they relate to system parameters. The model summarizes the main AC operating regimes for OECTs and helps predict the design parameters which dictate which frequency ranges are dominant for the different operating regimes.

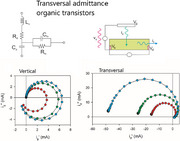

## 1 Introduction

Extensive research has been developed on OECTs in relation to diverse applications in bioelectronics, logic circuit components, and neuromorphic devices.^[^ ^1^, ^2^, ^3^, ^4^, ^5^, ^6^, ^7^, ^8^ ^]^ In the OECT, the channel is formed by an organic mixed ionic‐electronic conductor (OMIEC).^[^ ^9^, ^10^ ^]^ The electronic conductivity is controlled by a variable dopant density obtained by insertion and extraction of ions from an electrolyte and subsequent ion diffusion in the channel, while the compensating electronic carriers are injected from the drain and source contacts.^[^ ^11^, ^12^ ^]^ The combination of electrochemical, mixed ion‐electron conduction, and semiconductor properties imply complex device physics for the OECT and its variants.^[^ ^11^, ^13^ ^]^ Detailed characterization is needed to determine the kinetic properties of OECTs such as their switching times. In particular, hysteresis and memory properties of transistors are essential for neuromorphic functionalities.^[^ ^14^, ^15^, ^16^, ^17^ ^]^

Impedance spectroscopy (IS) is a very powerful tool for the characterization of electrochemical systems^[^ ^18^, ^19^, ^20^ ^]^ and semiconductor devices.^[^ ^21^ ^]^ Frequency domain analysis provides substantial advantages with respect to time domain for separating internal convoluted electrochemical processes, and for determining the capacitances, resistances, and inductors in the system.^[^ ^18^, ^19^, ^20^ ^]^ This method was developed and widely used in the early history of semiconductor transistors.^[^ ^22^ ^]^ However, for OECTs, there are only results considering partial aspects.^[^ ^23^, ^24^, ^25^, ^26^, ^27^, ^28^, ^29^, ^30^ ^]^ The impedance spectroscopy of ion‐controlled transistors has shown its value in identifying transport features^[^ ^23^ ^]^ and for developing sensing properties.^[^ ^31^, ^32^ ^]^ To the best of our knowledge, however, there is no systematic treatment of the IS of ion‐controlled transistors based on transport and conservation equations and including the ion diffusion process.

Obtaining a general framework that can be adapted to specific applications is the goal of this paper. There are many opportunities to use OECTs for impedance sensing when the impedances are very high, rendering the output currents low, as a tool for amplifying the output signal of the sensor. Some examples include sensing of cell layers^[^ ^32^, ^33^ ^]^ and amplifying electrode‐based biosensors.^[^ ^34^ ^]^ However, using and optimizing OECTs for applications with small signal detection requires a good understanding of their small signal response. Our model summarizes the main alternating current (AC) operating regimes for OECTs and helps predict the design parameters that dictate which frequency ranges are dominant for the different operating regimes.

**Figure 1:**
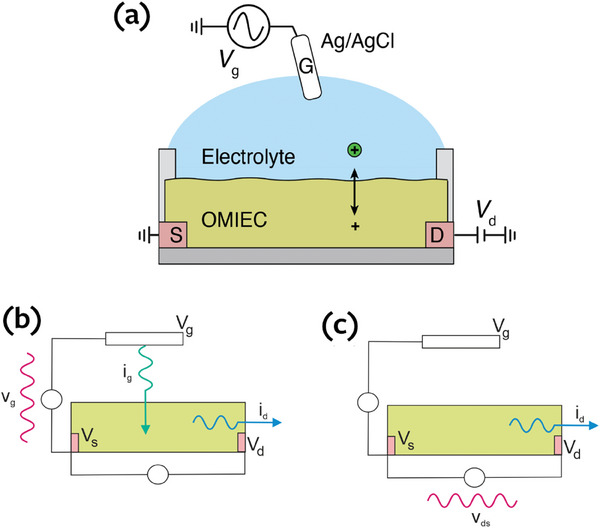
a) Schematic experimental setup (gate electrode not drawn to scale) of measurement. b) Scheme of the measurement of AC currents *i_g_*, *i_d_* in response to applied AC voltage *v_g_*, *v_ds_* in an ion‐controlled transistor. The uppercase letters *V_g_*, *V_s_*, *V_d_* indicate the stationary point of measurement. Configuration to measure the vertical admittance *Y_v_* = *i_g_* /*v_g_* and the transversal admittance *Y_tv_* = *i_d_* /*v_g_*. (c) Measurement of the horizontal admittance *Y_h_* = *i_d_* /*v_ds_*.

Experience has been accumulated over the last century of IS of two‐contact device systems. However, the OECT shows the peculiar property that it is a three‐contact device, leading to different possible ways to measure the impedances, as indicated in **Figure** **1**, as explained in Section 2.

The spread of current from drain to source and the vertical insertion process make the transistor current‐voltage analysis a double transmission line problem (one horizontal, and one vertical, intrinsically coupled) which is quite demanding to solve, both in field‐effect and double‐layer transistors^[^ ^35^, ^36^, ^37^ ^]^ and in OECTs.^[^ ^24^, ^25^, ^38^, ^39^, ^40^ ^]^ Fortunately, there are many studies of transient current using a simplified homogeneous approximation,^[^ ^23^, ^24^, ^41^, ^42^, ^43^ ^]^ based on the Bernard and Malliaras (BM) model.^[^ ^44^ ^]^ Here we use a similar approach, expanding the insights of a recent model,^[^ ^45^, ^46^ ^]^ to develop consistent and general admittance expressions for the ion‐controlled transistors.

We start with an introduction to the basic conventions of impedance/admittance properties in Section 2. In Section 3 we describe the features of elementary behavior of capacitive and inductive circuits. We then analyze the features of the impedance for ion intercalation by diffusion. Thereafter we derive the vertical and transversal admittances.

In Section 4 we explore the current ratio method for extracting mobility, that considers measurements at several frequencies to extract the mobility, rather than relying on only a single frequency. We show an extension of this method based on the relationship of transversal to vertical admittance. In Section 5 we present experimental results on frequency modulated currents in a Poly(3,4‐ethylenedioxythiophene)‐poly(styrenesulfonate) (PEDOT:PSS) OECT and discuss the spectral properties at different stationary points. In Section 6 we summarize the general transmission line model including the diffusion transport, that provides the current in any time‐dependent situation.^[^ ^45^ ^]^ Then in Section 7 we show that the model accounts for the spectral features of the experimental results, including the modifications caused by the change of stationary drain voltage *V_d_*. We finish with some conclusions.

## 2 Model and Definitions

**Figure 2:**
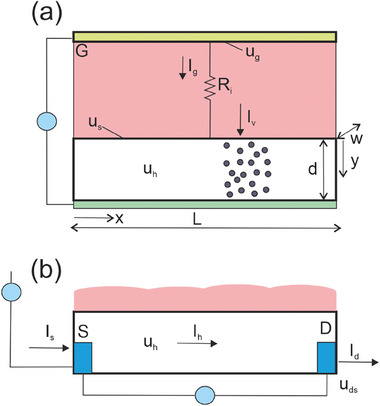
Scheme of the OECT. The pink zone is the electrolyte, yellow the gate contact, white the channel. a) The measurement of the ion insertion current between gate electrode and the substrate of the film (green). b) The measurement of the drain‐source current.

The main physical processes and the geometric features that we address for the measurement of the type of Figure 1a are outlined in **Figure** **2**.

### 2.1 Carrier Distribution

The effective cation density *z* depends on the concentrations of intercalated cations *m* and anions *a*

(1) z=m−a

By local electroneutrality, the local hole density is

(2) $$p=p_{0}\;-z$$

where *p*_0_ in an intrinsic density due to doping. Assuming a nearly homogeneous distribution in the vertical direction we define a density per unit length.

(3) $$Z=w\int_{0}^{d}z\;dy$$

Hence we define the linear densities

(4) $$A\left(x\right)=w\int_{0}^{d}a\;dy$$

(5) $$M\left(x\right)=w\int_{0}^{d}m\;dy$$

(6) Z=M−A

and similarly

(7) $$P=w\;\int_{0}^{d}p\;dy=P_{0}\;-Z$$

We assume an accumulation transistor where *p*_0_ =  *m*  =  0. The case of the depletion transistor can be treated similarly as commented later. The electroneutrality condition is

(8) p=a

### 2.2 Stationary Operation Point

The ion density in the film is a function of the electrochemical potential. If ion exchange is possible across the electrolyte‐film interface, the electrochemical potential inside the film equilibrates to the electrochemical potential in the electrolyte. For a stationary gate voltage *V_g_* in Figure 2a the concentration equilibrates to

(9) $$A=A_{eq}\;\left(V_{g}\right)$$

where *A_eq_* is the equilibrium thermodynamic function of the ionic concentration.^[^ ^47^, ^48^, ^49^, ^50^, ^51^ ^]^ The function *A_eq_* can have contributions from both ions, electronic carriers, and their interactions.

Consider a stationary drain‐source voltage *V_ds_*. The drain current in Figure 2b is

(10) $$I_{d}^{st}=-q\;\mu_{p}\;A\frac{V_{ds}}{L}$$

provided that *V_ds_* is small and *A*(*x*) is homogeneous. Here, *q* is the elementary charge, μ_*p*_ the hole mobility, *L* the channel length. With respect to the homogeneous volume concentration of ions and holes, *a*_0_, Equation (10) is

(11) $$I_{d}^{st}=-q\;\frac{w\;d\;V_{ds}}{L}\;\mu_{p}\;a_{0}$$

**Figure 3:**
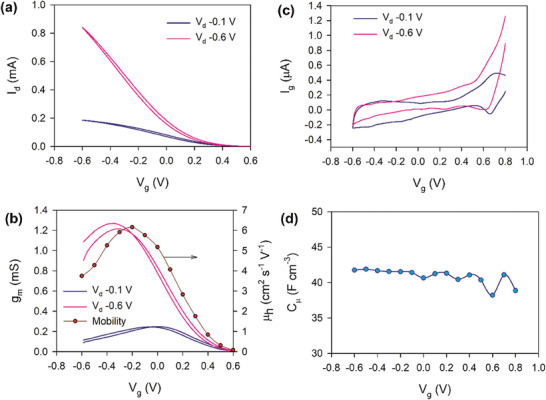
Characteristics of the OECT at two different drain voltages. a) Transfer curve, b) transconductance and mobility, c) gate current versus voltage, and d) chemical (volume) capacitance (obtained using the AC gate voltage method).

For the experimental results, we use an OECT device with PEDOT:PSS as the channel material, gold source and drain contacts, and a silver/silver chloride (Ag/AgCl) gate electrode with channel dimensions L = 800 µm, w = 100 µm. In the AC measurements, the gate voltage oscillates sinusoidally with an amplitude 0.025 V. The preparation of the devices and measurement procedures are explained in Section 5. The transfer curves and capacitance of the OECT studied here are shown in **Figure** **3**.

The Figure 3a shows that the pair of voltages (*V_g_*, *V_ds_*) determines a stationary point, which establishes different variables such as the transconductance and mobility in Figure 3b.

### 2.3 Small Signal AC Measurement

To study dynamical properties of the transistor we apply perturbations that depart from one stationary point. For example, we can study transient behaviors. Then variable voltages will occur in the system denoted *u_g_*, *u_d_* … Note in Figure 2 that *u_g_* is the gate voltage, *u_s_* is the potential at the outer surface of the OMIEC, and *u_h_* is the internal potential in the OMIEC.

In this paper we aim to study small perturbation methods. It means that we will stay close to a stationary point, and the model equations can be linearized. The small perturbation voltages are named *v_g_*, *v_d_* … and small perturbation currents are denoted *i_k_*. The vertical small perturbation gate current is *i_g_* and the drain current is *i_d_*.

One can study the small perturbation transient behavior in the time domain. The applied gate voltage is *u_g_* = *V_g_*  + *v_g_* (*t*) with small amplitude |*v_g_* | and the measured drain current is $I_{d}=I_{d}^{st}+i_{d}\left(t\right)$.^[^ ^46^ ^]^ But in this paper the main goal is to apply impedance analysis, by measuring in the frequency domain.

The IS measurement consists of establishing equilibrium at a given operation point of the gate voltage *V_g_* and drain‐source voltage *V_ds_*, and performing there a small perturbation current‐voltage measurement at varying angular frequency ω, that corresponds to the frequency *f*  =  ω/2π. Hereafter *v_g_*, *i_g_*, and all low‐case linear variables, also indicate Laplace domain variables, i.e., the Laplace transform of *v_g_* (*t*) is denoted *v_g_* (ω), which is a complex number, and similarly *i_d_* (*t*) → *i_d_* (ω).

For a couple of modulated voltage/current variables the AC impedance is defined as

(12) $$Z\;\left(\omega \right)=\frac{v\left(\omega \right)}{i\left(\omega \right)}\;$$

with the correspondent admittance

(13) $$Y\;\left(\omega \right)=\frac{1}{Z\left(\omega \right)}\;$$

Since the current *i*(ω) in response to *v*(ω) develops a phase difference, *Z*(ω) is a complex number. We represent impedances in the complex plane as *Z*  = *Z*′  + *jZ*′′ where $j=\sqrt{-1}$, and the admittance as *Y*  = *Y*′  + *jY*′′. Another important representation is given by the modulus $\left|Y\right|=\sqrt{YY^{*}}$ and the phase angle 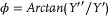.

Since *Z*(ω) and *Y*(ω) are the solution to a linearized model, they can be represented by an equivalent circuit.

As indicated in Figure 1a the vertical admittance is defined as

(14) $$Y_{v}=\frac{i_{g}}{v_{g}}\;$$

The transversal admittance *Y_tv_* is a three‐contact quantity, that does not follow the conventional definition of an impedance as a two‐port transfer function. The transversal admittance corresponds to the general idea of a *transfer admittance*, where the input is a voltage source and the output is the current through another branch of the circuit.^[^ ^52^ ^]^ In particular we adopt the measurement of the drain current *i_d_* (ω) under applied gate voltage *v_g_* (ω),

(15) $$Y_{tv}=\frac{i_{d}}{v_{g}}\;$$

The function *Y_tv_* provides important information about the transistor dynamic behavior, and we aim to provide a model description and validation experiments in this paper.

Figure 1b shows the horizontal admittance,

(16) $$Y_{h}=\frac{i_{d}}{v_{ds}}\;$$

This is a conventional two‐port admittance that is not investigated in this paper although some comments are provided in Section 7.6.

## 3 Modeling Transistor Admittances

To illustrate the meaning and interpretation of admittances in an OECT, in the next subsections, we derive the main properties starting from intuitive considerations. These models will be developed in detail in a general framework outlined in Sections 5 and 6.

### 3.1 Charging the Organic Layer

The ionic charge in the channel can be modulated by the gate voltage, by insertion and extraction of ions in the channel. When a voltage is applied to the gate in Figure 2a, which has an electronically conductor substrate in the channel, only ions can enter the channel film from the liquid phase, since the electrolyte is an electronic insulator. Charge‐compensating electronic carriers can enter from the substrate electrode (or from drain and source electrodes in a transistor), which is a barrier to ions. Therefore, the response to a change of *V_g_* is a transient current, shown in Figure 3c, while stationary *I_g_* current is not possible.

Let us analyze the charging of the OMIEC in Figure 2a. The total electrical charge at equilibrium is

(17) $$Q=qL\;A_{eq}\left(V_{g}\right)$$

If we apply a small step of the voltage there will be a step of charge inserted or extracted.

(18) $$\Delta Q=qL\frac{dA}{du}\;\Delta V$$

The chemical capacitance *C*_μ_ is the derivative of the concentration of a species with respect to the electrochemical potential.^[^ ^11^, ^53^, ^54^ ^]^

(19) $$C_{\mu }=-qL\;\frac{dA_{eq}}{du}=Lwd\;q\left|\frac{da_{eq}}{du}\right|$$

The chemical capacitance is proportional to the volume of material. The passed charge can be expressed:

(20) $$\Delta Q=-C_{\mu }\Delta V$$

The denomination of a chemical capacitance appears in the transmission line representation of the impedance of mixed ionic‐electronic conductors^[^ ^55^ ^]^ and in the analysis of electrochemical solar cells.^[^ ^56^ ^]^ It is the same quantity as the diffusion capacitance for electrons in semiconductors,^[^ ^57^ ^]^ the redox capacitance of organic films,^[^ ^58^ ^]^ and the intercalation capacitance for ions in electrochemical insertion.^[^ ^59^, ^60^ ^]^ It is often denominated “volume capacitance” in the literature of OECTs.

**Figure 4:**
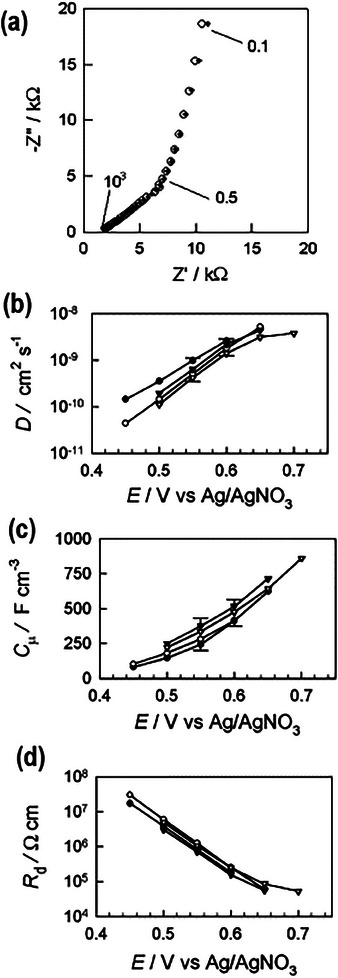
Results of electrochemical impedance measurements of polydicarbazole films at different steady‐state potentials (film thickness 400 nm). a) Comparison between experiment and fit (+) for the spectra taken at 0.5 V versus Ag/AgNO_3_. Some frequencies are marked (in Hz). Diffusion frequency 1/2πτ_*d*_ ≈ 0.5 Hz. b) Chemical diffusion coefficient of the ions, c) chemical capacitance, and d) diffusion resistance, for four different film thickness. Reproduced from.^[^ ^64^ ^]^

Due to the restriction of electroneutrality in Equation (8), the chemical capacitance in Equation (19) can obtain contributions from both the ionic and electronic terms, depending on the respective densities of states.^[^ ^51^, ^61^, ^62^, ^63^ ^]^ The chemical capacitance of the PEDOT transistor shown in Figure 3d is nearly constant. **Figure** **4**c shows the chemical capacitance of polydicarbazole films, that increases exponentially with the voltage.

Consider the charging by a gate voltage during a time d*t*. The current is obtained from the change of ionic density

(21) $$I_{g}=-qL\frac{dA}{dt}$$

The passed charge relates to the voltage inside the organic layer as *A*(*u_h_*). Therefore, we can write

(22) $$I_{g}=C_{\mu }\;\frac{du_{h}}{dt}$$

The series resistance *R_i_* in Figure 2a establishes a voltage difference *u_g_* − *u_s_*. Therefore

(23) $$I_{g}=\frac{1}{R_{i}}\;\left(u_{g}-u_{s}\right)$$

To complete the circuit one more relation is needed to connect *u_s_* and *u_h_*. This will be established in Section 3.3 by the diffusion equations. For the moment we assume that the surface potential is the same as the internal potential, *u_s_* = *u_h_* . Hence

(24) $$I_{g}=\frac{1}{R_{i}}\;\left(u_{g}-u_{h}\right)$$

To the system (21, 23) we apply the small perturbation in the Laplace domain, with the Laplace variable *s*  =  *j*ω. Equation (21) gives

(25) $$i_{g}=Y_{\mu }\;v_{h}$$

in terms of the admittance of the chemical capacitance, namely

(26) $$Y_{\mu }=C_{\mu }\;s$$

From Equation (23)

(27) $$i_{g}=\frac{1}{R_{i}}\;\left(v_{g}-v_{h}\right)$$

Let us define the characteristic frequency

(28) $$\omega_{RC}=\frac{1}{R_{i}\;C_{\mu }}\;$$

We solve Equations (25) in 25 and ((25), (27)) and find the admittance

(29) $$Y=\frac{i_{g}}{v_{g}}=\frac{C_{\mu }s}{1+\frac{s}{\omega_{RC}}}\;$$

**Figure 5:**
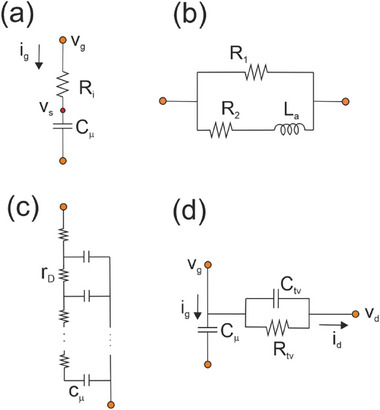
Basic equivalent circuit models. a) The vertical impedance of the transistor including *RC* elements. b) Inductive circuit. c) Transmission line diffusion impedance of a finite layer with blocking boundary condition. d) The transversal impedance of the transistor.

The model is represented as the equivalent circuit in **Figure** **5**a.

**Figure 6:**
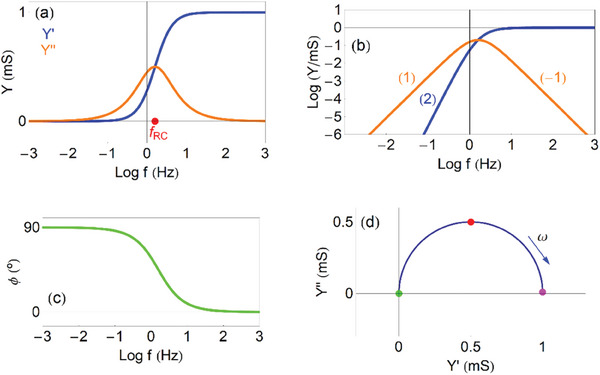
Admittances of the capacitive circuit. a) Real and imaginary parts of the admittance as function of frequency, indicating the frequency *f_RC_*. b) Vertical axis log representation of (a), indicating the slopes of the lines. c) Phase angle as function of frequency. d) Complex plane plot representation of the admittances, indicating the points ω  =  0 (green), ω_*RC*_ =  10 *rad*/*s* (red), ω  =  ∞ (purple), and the direction of increasing frequency. *R_i_* =  1 *k*Ω cm, *C*_μ_ =  0.1 *mF*.

Now we plot the admittance function to identify the dominant trends of the model, for recognizing this process in experimental results. In **Figure** **6**a we show the real and imaginary parts of the admittance as a function of the frequency. The admittance (29) is capacitive at low frequencies, and becomes a conductance at high frequencies, as follows

(30) $$Y_{v}\approx j\omega C_{\mu }\;\omega \ll \omega_{RC}$$

(31) $$Y_{v}\approx \frac{1}{R_{i}}\;\omega \gg \omega_{RC}$$

These two branches are observed at low and intermediate frequencies in Figure 6a. The real part of the admittance *Y*′ indicates a conductance. The *Y*′ starts from 0 at low frequency. We already mentioned that the stationary *I_g_* =  0, as the circuit is blocking the current. But the transient current increases with frequency, up to the maximum conductance 1/*R_i_* at high frequency. When the *Y*′ increases between two plateaus, there is a peak of the *Y*′′. This is a very general feature, rooted in the Kronig‐Kramers transform properties.^[^ ^65^, ^66^, ^67^ ^]^ The frequency ω_*RC*_ marks the maximum of 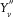 and the transition between the two regimes in Equations ((30), (31)).

In the vertical log plot of Figure 6b we observe the linear wings of the capacitive peak, with slopes +1 and ‐1, that enable to recognize this feature in measurements. The phase angle in Figure 6c is initially capacitive (90°) and becomes resistive (0°) at high frequency. The complex plane plot in Figure 6d shows a positive arc.

The model in Figure 5a and Figure 6 is our basic model for the vertical impedance of the OECT. However, it is possible in theory to eliminate the series resistance *R_i_*, so that the minimal feature of the vertical impedance is the chemical capacitance. In other approaches^[^ ^68^ ^]^ the volume capacitance is interpreted as the double layer charging.

### 3.2 Inductive Process

To complete the analysis of the impedance of basic elements we discuss the inductive circuit of Figure 5b, that contains a chemical inductor, represented by the element *L_a_* and a series resistance *R*_2_. This circuit finds numerous applications in memristors and systems with memory effects.^[^ ^69^ ^]^

The admittance of this circuit is

(32) $$Y=\frac{1}{R_{1}}+\frac{1}{R_{2}+s\;L_{a}}$$

**Figure 7:**
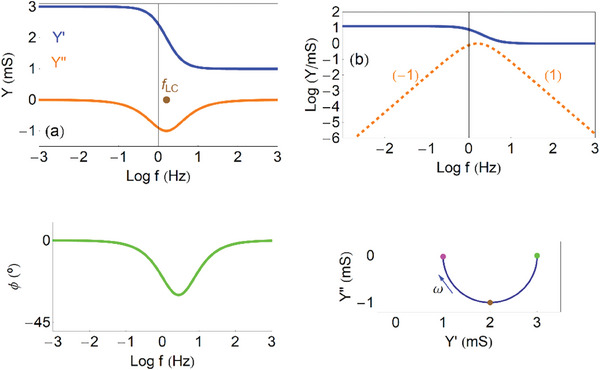
Admittances of the inductive circuit. a) Real and imaginary parts of the admittance as function of frequency, indicating the frequency *f_RL_*. b) Vertical axis log representation of (a), indicating the slopes of the lines (the dashed line is *Log*(− *Y*′′)). c) Phase angle as function of frequency. d) Complex plane plot representation of the admittances, indicating the points ω  =  0 (green), ω_*RL*_ =  10 *rad*/*s* (brown), ω  =  ∞ (purple), and the direction of increasing frequency. *R*_1_ =  1 *k*Ω, *R*_2_ =  5 *k*Ω, *L_a_* =  0.05 *mF*.

As shown in **Figure** **7**a the *Y*′ makes a transition from the stationary conductance 1/*R*_1_ + 1/*R*_2_ to the high frequency value 1/*R*_2_. The change occurs at the characteristic frequency

(33) $$\omega_{LC}=\frac{R_{2}}{L_{a}}\;$$

The *Y*′′ traces a negative peak, hence the conductance decreases with frequency. The inductor can be viewed as a negative capacitance, with inverted slopes with respect to the capacitive peak, Figure 7b. The phase angle takes negative values, and the complex plane representation is an arc in the fourth quadrant.

### 3.3 Diffusion

We considered previously an instantaneous charging of the channel film when a voltage is applied to the surface. However, when the surface potential *u_s_* is modified, the ions take some time to establish equilibrium of the internal voltage *u_h_*. This is an intercalation process similar to that occurring in batteries, and it is well described by diffusion.^[^ ^70^ ^]^ The diffusion charging of ions into organic layers has been amply studied, starting with the conducting polymer films,^[^ ^64^, ^71^, ^72^ ^]^ and also in the organic transistors.^[^ ^73^, ^74^ ^]^ Here we review the main elements of impedance interpretation.

Since *a* is the anion density at a point inside the film, the ion transport in Figure 2a is established by the diffusion equation that relates the flux *J_v_* with the gradient of concentration via the diffusion coefficient *D_ion_*

(34) $$J_{v}=-D_{ion}\frac{\partial a}{\partial y}$$

The conservation equation is

(35) $$\frac{\partial a}{\partial t}=-\frac{\partial J_{v}}{\partial y}$$

The boundary condition *J_v_* (*y*  =  *d*) =  0 expresses ion blocking at the bottom electrode.

The Equations (34 and 35) for ion diffusion determine the transient insertion process provided that the electronic density is enough to shield electrical fields in the vertical direction. In general, the vertical transport may contain both ionic and electronic components, according to the doping conditions,^[^ ^11^, ^12^, ^75^ ^]^ but here we consider the dominance of ionic transport, which is the species that crosses the top interface in Figure 2a.

The diffusion impedance can be obtained solving (34,35) with the result^[^ ^76^ ^]^

(36) $$Z_{dif}=R_{D}\;{\left(s\;\tau_{d}\right)}^{-0.5}\mathrm{Cotanh}\left[{\left(s\;\tau_{d}\right)}^{0.5}\right]$$

The ion diffusion time across the layer, τ_*d*_, is

(37) $$\tau_{d}=\frac{d^{2}}{D_{ion}}\;$$

and the diffusion resistance *R_D_* is given by

(38) $$R_{D}=\frac{\tau_{d}}{C_{\mu }}=\frac{d}{Lw}\;\frac{1}{D_{ion}q\;\left|da/du\right|}$$

The diffusion resistance is proportional to the film thickness and inversely proportional to the top area.

**Figure 8:**
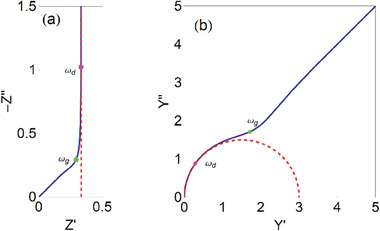
Impedance of diffusion in a restricted layer. a) Complex plane plot impedance spectra. The blue line is the finite transmission line model, and the red line is the *R_d_C*_μ_ series model. The points indicate characteristic frequencies, ω_*d*_ =  1/τ_*d*_, and the frequency of the ankle, ω_*g*_ =  (π^2^/2) ω_*d*_.

The diffusion impedance for a blocking boundary is shown in **Figure** **8**a, where the usual 45° (Warburg) line is observed. The associated transmission line equivalent circuit is represented in Figure 5c.^[^ ^77^, ^78^ ^]^ This is the impedance of ion intercalation in polymer films, that is well understood, and representative measurements are shown in Figure 4.^[^ ^64^ ^]^ The fit of the impedance data with the model enables the determination of the chemical diffusion coefficient, chemical capacitance, and diffusion resistance. In the distributed capacitance in a transmission line, the capacitance is related to the local concentration of charge carriers.^[^ ^57^ ^]^

Normally, in the measurement of disordered materials, the diffusion and capacitance lines are inclined less than 45° and 90°, as in Figure 4a, and the use of constant phase elements (CPE) becomes necessary.^[^ ^79^, ^80^ ^]^ In other cases,^[^ ^81^, ^82^ ^]^ the impedance spectra becomes distorted by charge transfer elements, that can be represented by the boundary conditions of the transmission line.^[^ ^83^ ^]^

The low‐frequency approximation of (36) is^[^ ^76^ ^]^

(39) $$Z_{d}=R_{d}+\frac{1}{C_{\mu }s}$$

where

(40) $$R_{d}=\frac{R_{D}}{3}\;$$

The model (39) is an *RC* series circuit, as in Figure 5a. The complex plane representation of Equation (39) is shown in the red line in Figure 8. We note that the simpler model is accurate at frequencies lower than

(41) $$\omega_{d}=\frac{1}{\tau_{d}}\;$$

Therefore, the 45° line associated to semi‐infinite diffusion is lost in the simplified model. A full discussion of the time domain properties is provided in ref. [46]

In correspondence with the simpler model we introduce an approximation to the diffusion equations.^[^ ^84^ ^]^ In the flux equation, we replace the local gradients by the difference of internal and external concentrations

(42) $$J_{v}\;\left(0\right)=-D_{ion}\frac{\partial a}{\partial y}\approx -\frac{D_{i}}{d}\left[a\left(d\right)-a\left(0\right)\right]$$

The concentrations are fixed by the internal and surface voltages, *u_h_*, *u_s_* respectively, that are different when there occurs a diffusion delay. Applying the averaging in Equation (4)

(43) $$J_{v}\;\left(0\right)=-\frac{1}{w\;\tau_{d}}\left[A\left(u_{h}\right)-A\left(u_{s}\right)\right]$$

The vertical flux of anions at the film/electrolyte interface is *J_v_* (*y*  =  0). The vertical current entering a small volume element of width *dx* is

(44) $$I_{v}=-q\;w\;J_{v}\left(y=0\right)$$

From Equation (31)

(45) $$I_{v}=-\frac{q}{\;\tau_{d}}\left[A\left(u_{s}\right)-A\left(u_{h}\right)\right]$$

The charging of the chemical capacitance inside the layer is described by Equation (21). The total gate current is *I_g_* =  *LI_v_*. Combining Equations (21) and (45) we have

(46) $$\tau_{d}\frac{dA}{dt}\left(u_{h}\right)=A\left(u_{s}\right)-A\left(u_{h}\right)$$

Let us assume *u_s_* = *u_g_* (neglecting external resistances). The small perturbation and Laplace transform of Equations (21) and (46) gives, respectively

(47) $$i_{g}=C_{\mu }\;s\;v_{h}$$

(48) $$\tau_{d}s\;v_{h}=v_{g}\;-v_{h}$$

We thus obtain the admittance

(49) $$Y=\frac{1}{R_{d}+\frac{1}{C_{\mu }s}}\;$$

This is the same model as Equation (29). But now the impedance is due to the ionic processes inside the film. The impedance *Z_d_* in Equation (36) is obtained using the approximation (43), instead of the full diffusion model. This simplification produces several deviations of the impedance model, that can be observed in the Figure 8: the 45° line is suppressed, and the apparent low frequency diffusion resistance *R_d_* is decreased by a factor 3. However, the use of (43), and the associated impedance *Z_d_*, provides important rewards for the interpretation of the transversal impedance, as we explain in Section 3.5.

### 3.4 The Transient Horizontal Current

We now address the processes of the horizontal current shown in Figure 2. The stationary current has been written in Equation (11). The horizontal electronic flux is

(50) $$J_{h}\;\left(x,y\right)=-p\left(x,y\right)\mu_{p}\frac{du_{h}}{dx}$$

The horizontal current is

(51) $$I_{h}\;\left(x\right)=q\;w\;\int_{0}^{d}J_{h}\;dy$$

hence, applying electroneutrality condition,

(52) $$I_{h}=-q\;A\;\mu_{p}\frac{du_{h}}{dx}$$

Hereafter we consider that the applied drain‐source voltage does not cause significant horizontal inhomogeneity of charge *A*. The electric field in the channel can be stated as *u_h_*/*dx*  = *V_ds_*/*L*. Hence

(53) $$I_{h}\;\left(x\right)=-\frac{q\theta L}{\tau_{e}}\;A\left(x\right)$$

where

(54) $$\tau_{e}=\frac{L^{2}}{\mu_{p}\left|V_{ds}\right|}\;$$

is the electronic transit time, and

(55) $$\theta =\frac{V_{ds}}{\left|V_{ds}\right|}\;$$

is the sign of the driving electrical field.

When the organic layer is charged with ions by the vertical current formed by *u_g_*, a transient electronic current occurs, injected from drain and source electrodes, to satisfy electroneutrality. This process of charging can be described by the equation developed by BM model,^[^ ^44^ ^]^ that we express as follows

(56) $$I_{d}=-\frac{q\theta L}{\tau_{e}}\;A-q\;L\;f_{B\;}\frac{dA}{dt}$$

This equation is justified below.^[^ ^45^ ^]^ The current fraction factor 0 < *f_B_* < 1 indicates the fraction of the charging electronic current that is injected at the drain electrode.

Equation (52) is not a general transport equation. By the electroneutrality condition, there is a shielding effect on the internal field, and the transport may be driven by hole diffusion. These are complex questions about the transport model that have been addressed recently.^[^ ^11^ ^]^ Hereafter we adopt the classical simplified view based on Equation (50), as the main problem we face is the coupling of horizontal and vertical currents.

### 3.5 The Transversal Admittance

We calculate the small perturbation current associated to Equation (56) and we obtain

(57) $$i_{d}=\frac{\theta C_{\mu }}{\tau_{e}}\;v_{h}+f_{B}C_{\mu }\frac{dv_{h}}{dt}$$

In the Laplace domain

(58) $$i_{d}=C_{\mu }\;\left(\frac{\theta }{\tau_{e}}+f_{B}s\right)v_{h}$$

Note that Equation (58) is the expression of BM model^[^ ^44^ ^]^ for a small AC perturbation.

Let us introduce the transversal admittance parameters. The transversal electronic transport resistance is

(59) $$R_{tv}=\theta \;\frac{\;\tau_{e}}{C_{\mu }}=\frac{\theta \;L^{2}}{C_{\mu }\mu_{p}\left|V_{ds}\right|}=\frac{L}{wd}\;\frac{\theta }{q\mu_{p}\left|V_{ds}\right|\;\left|da/du\right|}$$

The transversal resistance is proportional to channel length and inversely proportional to the cross section *wd*. Equation (59) is obtained by modulation of the carrier density, in distinction to the channel electronic resistance that is described in Section 7.6.

The transversal electronic capacitance is

(60) $$C_{tv}=f_{B}\;C_{\mu }$$

In Equation (60) *C*_μ_ is the chemical capacitance of ions, that is charged by the vertical current. The *C_tv_* is the chemical capacitance of holes in the channel (by modulation of the gate voltage). It is related to *C*_μ_ due to the electroneutrality condition, but the factor *f_B_* indicates the part of the total chemical capacitance that contributes to the drain current.

The effective relaxation time of the electronic channel is

(61) $$R_{tv}\;C_{tv}=\theta f_{B}\;\tau_{e}=\theta \tau_{h}$$

where

(62) $$\tau_{h}=f_{B}\;\tau_{e}$$

We define the frequency

(63)

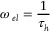




Equation (58) can be written

(64) $$i_{d}=Y_{el}\;v_{h}$$

where the electronic admittance is

(65) $$Y_{el}=\frac{1}{R_{tv}}+C_{tv}s=\frac{1}{R_{tv}}\;\left(1+\frac{s}{\omega_{el}}\right)$$

In Figure 5d we draw the branch of horizontal transport that represents Equation (65).

To establish the transversal admittance *Y_tv_* = *i_d_* /*v_g_* in Equation (15), a relation *v_h_* (*v_g_*) is needed. We have previously shown that the gate current charges the chemical capacitance by the admittance *Y*_μ_, Equation (26). Thus *i_g_* charges the vertical admittance as follows

(66) $$i_{g}=Y_{v}\;v_{g}$$

We obtain the relation

(67) $$v_{h}=\frac{Y_{v}}{Y_{\mu }}\;v_{g}$$

Combining Equation (64) and (67) we obtain

(68) $$Y_{tv}=\frac{Y_{v}}{Y_{\mu }}\;Y_{el}$$

This is a main result that expresses the transversal admittance in terms of the different parts that compose it. The factor *Y_v_*/*Y*_μ_ modulates the horizontal impedance *Y_el_*. *Y_v_*/*Y*_μ_ depends on the elements in series.

It is important to remark that the horizontal branch in Figure 5d, related to Equation (65), starts from the internal voltage *v_h_*, that is placed between the diffusion resistance and the diffusion capacitance. The *v_h_*, viewed as an electrochemical potential, tracks the amount of charge in the diffusion layer (the charging of the chemical capacitor), as in all diffusion problems.^[^ ^85^ ^]^ By the approximation (45), we obtain the separation of *R_d_* and *C*_μ_ which makes visible the connection of the horizontal branch. We remark that the formula (68) is only valid if the potential *v_h_* is homogeneous inside the organic layer. It therefore relies on the simplified diffusion model of Equation (45).

The coupling of vertical and horizontal elements in the equivalent circuit is much more complicated if the full transmission line of Figure 5c is used, as then *v_h_* is varying in the vertical direction. Therefore Equation (45) enables significant insight to the structure of the transversal impedance, admittedly with some penalty in the fidelity of the overall impedance model.

**Figure 9:**
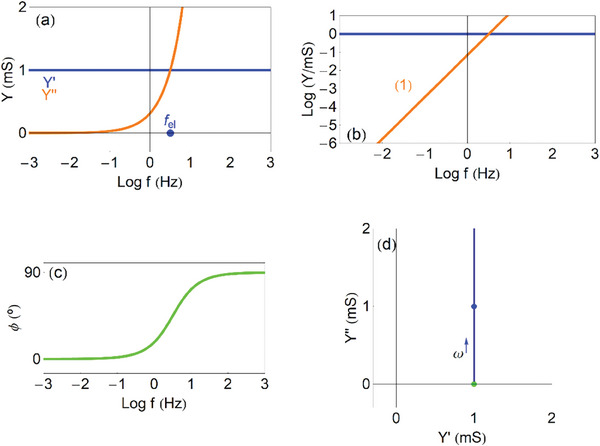
The transversal admittance. The vertical admittance is the chemical capacitance, and the horizontal transport/conservation equation is the BM model. a) Real and imaginary parts of the admittance as a function of frequency, indicating the frequency *f_el_*. b) Vertical axis log representation of (a), indicating the slopes of the line. c) Phase angle as function of frequency. d) Complex plane plot representation of the admittance, indicating the points ω_*el*_ =  20 *rad*/*s* (brown), ω  =  0 (green), and the direction of increasing frequency. *R_tv_* =  1 *k*Ω, *R_i_* =  0, *C*_μ_ =  0.1 *mF*, *f_B_* =  0.5.

Let us obtain the most elementary model of the transversal admittance, shown in Figure 5d, which provides the behavior shown in **Figure** **9**. For the time being let us assume no additional elements in the electrolyte so that *v_h_* = *v_g_* . The minimal element describing the organic layer is the charging of the chemical capacitance and we have *Y_v_* = *Y*_μ_ . Then

(69) $$Y_{tv}=Y_{el}=\frac{1}{R_{tv}}+C_{tv}s$$

In Figure 9 we observe that the conductance is independent of the frequency, 1/*R_tv_*, but the capacitive current increases with the frequency.

### 3.6 Depletion Mode Transistor

This is the case *A*  =  0, cation density *Z*  =  *M*, and hole carrier density *P*  = *P*_0_  − *M*, where *P*_0_ is the initial doping. The model equations are^[^ ^45^ ^]^

(70) $$I_{h}\;\left(L\right)=-\theta \frac{qL}{\tau_{e}}\left(P_{0}-M\right)+qfL\frac{dM}{dt}$$

(71) $$\tau_{d}\;\frac{dM}{dt}=M_{eq}\;\left(u_{g}\right)-M$$

In the small perturbation we obtain

(72) $$I_{d}=-\theta \frac{qL}{\tau_{e}}\;\left(P_{0}-M\left(u_{g0}\right)\right)+C_{\mu }\left(\frac{\theta }{\tau_{e}}v_{h}+f_{B}\frac{dv_{h}}{dt}\right)$$

where

(73) $$C_{\mu }=qL\frac{dM_{eq}}{du}$$

Comparing with Equation (55), we remark that the stationary expressions are different in accumulation and depletion, but the small perturbation method can be applied equally to both. Hence the forthcoming extensions apply equally to both types.

## 4 Determination of Device Parameters by Current Ratios

### 4.1 Extracting Mobility from the Low Frequency Response

The relation between the amplitudes of gate and drain currents is used to obtain the hole mobility.^[^ ^12^, ^23^ ^]^ The current ratio method for extracting mobility considers measurements at several frequencies to extract the mobility, rather than relying on only a single frequency. It is particularly useful for monitoring the effect of the gate potential (or the channel carrier concentration) on the carrier transport properties.

**Figure 10:**
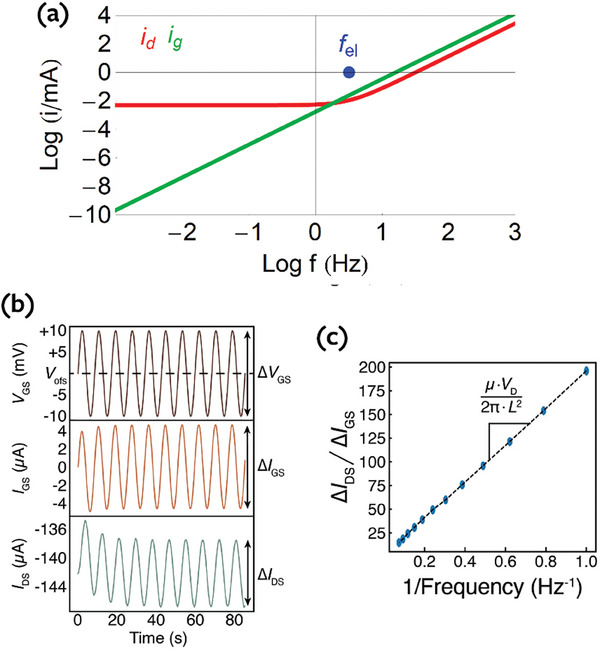
a) Representation of currents for the model of Figure 8, with Δ*V*  =  0.1 *V*. b) An oscillating sine wave voltage with amplitude of ± 10 mV is applied to the gate. The frequency‐dependent admittances can be extracted from the gate current and drain current divided by the amplitude of the gate voltage sine wave. c) The mobility can be extracted fitting a straight line to the ratio of drain current and gate current amplitudes as a function of the inverse frequency.

To extract the mobility, we measure the frequency response of the OECT over a range of frequencies as shown in **Figure** **10**b. The current ratio is obtained from the previous admittances as

(74) $$\frac{i_{d}}{i_{g}}=\frac{Y_{tv}}{Y_{v}}\;$$

From Equation (68) we obtain the general relationship

(75) $$\frac{i_{d}}{i_{g}}=\frac{Y_{el}}{Y_{\mu }}\;$$

Consider the specific model of Equation (69). We have

(76) $$\frac{i_{d}}{i_{g}}=\frac{C_{el}}{C_{\mu }}\;\left(1+\frac{\omega_{el}}{s}\right)$$

From Equation (76) we obtain the limit behavior

(77) $$\frac{i_{d}}{i_{g}}=\frac{\theta }{\tau_{e}\;j\omega }\;\left(\omega <<\omega_{el}\right)$$

At low frequency the currents run in 90° phase difference. This is shown in Figure 10a for the model of Figures 5d and 9. The separate currents are obtained from the admittances by Equations (14) and (15). We can write the relationship of the modulus of the currents in the standard form^[^ ^23^ ^]^

(78) $$\frac{\left|i_{d}\right|}{\left|i_{g}\right|}=\frac{\mu_{p}\left|V_{ds}\right|}{L^{2}}\;\frac{1}{2\pi f}$$

From the equivalent circuit in Figure 5d, we see that the charge density of the OECT is modeled as a capacitor *C*_μ_. This charge density also dictates the conductivity of the OECT channel, so we expect the change in channel current *I_d_* to be proportional to the current into or out of *C*_μ_, where a positive *I_g_* corresponds to cation insertion into the channel, which will decrease the conductivity, and a negative *I_g_* will pull cations out of the channel, increasing the conductivity. In other words, the time derivative of *I_d_* should be proportional to *I_g_*: *dI_d_*/*dt*∝*I_g_*, where the derivative of the sinusoidal gate current will be a cosine wave, or a sine wave with a 90° offset.

In the measurement results, we select only the data from the frequency range where there is a 90° phase offset between the gate and drain current responses. This 90° phase offset tells us that we are operating in a regime where the drain response is dominated by the changes in doping level. Then, we plot the current ratio versus the inverse frequency and fit the slope of the curve to extract mobility, as shown in Figure 10c.

### 4.2 Extracting the Current Fraction form the High‐Frequency Response

In the previously reported impedance matching technique,^[^ ^23^ ^]^ the standard method uses only the data points where there is a 90° phase offset. Let us also consider the high frequency region where 2π*f* > ω_*el*_. By Equation (76) we have

(79) $$\frac{\left|i_{d}\right|}{\left|i_{g}\right|}=\frac{C_{el}}{C_{\mu }}=f_{B}\;$$

This relationship implies that at high‐frequency *i_g_* (ω) and *i_d_* (ω) run parallel. This result is not reported previously to our knowledge. This behavior is clearly observed in the model simulations of Figure 10a. This new result occurs because at high frequency both currents are in phase: *i_g_* charges the chemical capacitance of ions and *i_d_* charges the transversal capacitance of electrons, which is reduced by *f_B_* with respect to *C*_μ_. The fractional parameter *f_B_* is commonly used in transient OECT models^[^ ^24^, ^43^, ^86^, ^87^ ^]^ but is difficult to estimate, so it is often used as a free parameter during fitting of the device response or assumed to be ½ for small drain biases. However, with our high‐frequency admittance model, the *f*_B_ parameter can be fit for a device and characterized as a function of the average carrier density in the channel by modulating the offset gate potential. This way, the dynamic *f*_B_ response can be characterized independently of other parameters determining the transient response of OECTs. We expect that *f*_B_ depends on several materials parameters, including the relationship between carrier concentration and both chemical capacitance and charge carrier mobility, the contact resistances, and the magnitude of the source‐drain bias compared to the gate bias.

## 5 Experimental Results

### 5.1 Experimental Methods

#### 5.1.1 Preparation of PEDOT:PSS Dispersion

The PEDOT:PSS dispersion was prepared by mixing Clevios PH1000 (Heraeus) with 6%v/v ethylene glycol and 1%v/v (3‐glycidyloxypropyl)trimethoxysilane, sonicating for 10 min, and then filtering through a 0.45 µm polyvinylidene fluoride syringe filter.

#### 5.1.2 Device Fabrication

Fabrication of organic electrochemical transistors for device characterization started with 10 min of sonication of borosilicate glass wafers (900 µm thick, double‐side polished Microchemicals) submerged in acetone followed by IPA then baked at 150 °C to remove any residual moisture. Gold contacts were patterned using metal lift off which consisted of coating with AZ nLoF 2035 negative photoresist (Microchemicals) (spin coating at 500 RPM for 5 s, acceleration of 1000 RPM s^−1^, followed by 3000 RPM for 45 s, acceleration of 8000 RPM s^−1^, then soft baked at 110 °C for 60 s) followed by UV exposure (60 mJ cm^−2^), a post exposure bake (110 °C for 180 s), and development in AZ 826 MIF (Microchemicals) for 30 s. The patterned wafer was coated with 5 nm titanium then 100 nm gold (E‐beam Evaporator, Kurt J. Lesker Company), and lift‐off was performed by submerging the wafer in acetone for 30 min followed by rinsing with IPA. The patterned metal‐coated wafers are then coated with a parylene bilayer by first treating the wafer with oxygen plasma for 60 s followed by submerging the wafer in a dilute silane solution (3%v/v A174 silane dissolved in 0.1%v/v acetic acid in deionized water) for 45 s to improve parylene adhesion to the wafer. The silane‐treated wafer is rinsed with ethanol and heated for 1 h at 75 °C then coated with a 2 µm layer of parylene (PDS 2010 Labcoter 2, Specialty Coating Systems) followed by coating with a soap surfactant layer (2%v/v/ Micro 90 soap in deionized water spin coated at 1000 RPM for 30 s and dried in air for 20 min) followed by a second deposition of a 2 µm layer of parylene. The trenches for depositing polymer channels are defined in the parylene bilayer with photolithography by coating with AZ10XT positive resist (Microchemicals) (spin coating at 3000 RPM for 45 s, acceleration of 8000 RPM s^−1^, soft bake at 115 °C for 120 s) followed by UV exposure (540 mJ cm^−2^) and developing in AZ 726 MIF developer (Microchemicals) for 10 min. Then, trenches are etched using reactive ion etching (recipe) and the wafers were diced with a diamond scribe and tile cutter tool. PEDOT:PSS was spin‐coated on the substrate with at a spin speed of 1000 RPM for 120 s. The PEDOT:PSS channel was defined by peeling off the top parylene layer using Kapton tape, leaving conducting polymer only in the patterned trench, followed by annealing at 120 °C for 20 min to dry and crosslink the film. A silicone well was defined using an adhesive backed silicone (McMaster‐Carr) to confine the electrolyte.

#### 5.1.3 Device Characterization

Electrochemical transistors were characterized using a Keysight B2902A Source‐Measure Unit controlled custom Python code. The electrolyte was 100 mM aqueous NaCl.

#### 5.1.4 PEDOT:PSS Thickness Measurements

Film thicknesses were measured using a DekTak XT Profilometer with a scan rate of 17 µm s^−1^ and a stylus force of 1 mg. The sample was measured in 6 locations and averaged to obtain a thickness of 224 ± 10 nm for the OECT channels.

### 5.2 Results

**Figure 11:**
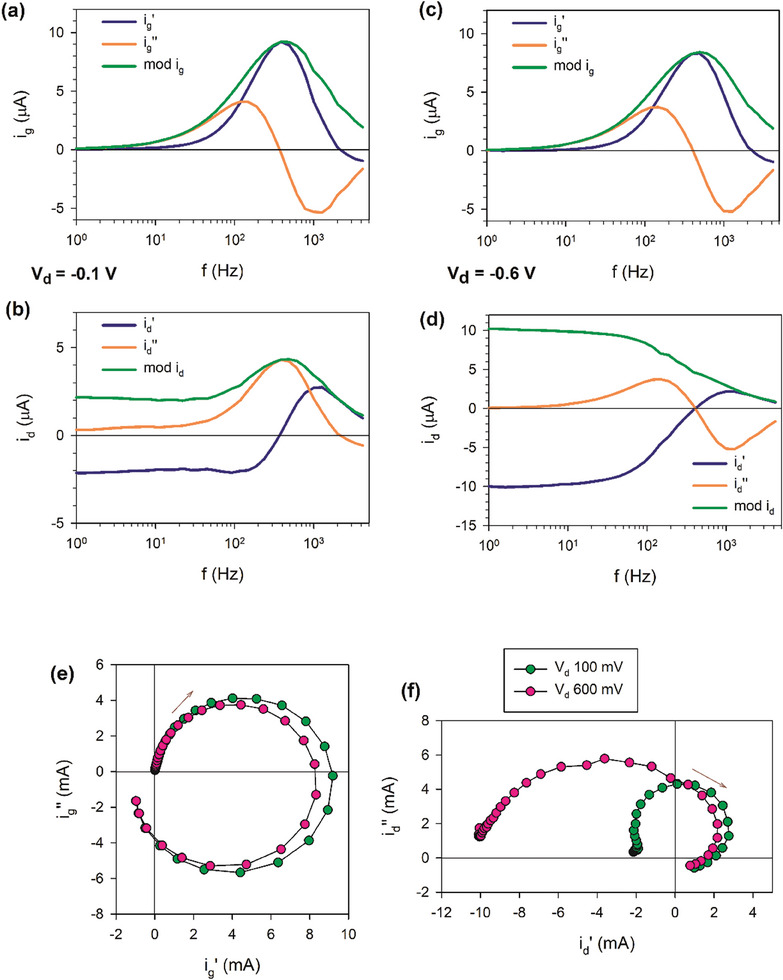
Experimental results for a transistor of channel dimensions L = 800 µm, w = 100 µm. Gate (*i_g_*) and drain (*i_d_*) complex currents as a function of frequency (a–d) for a gate voltage *V_g_* =   − 0.1 V. *mod* *i_k_* = |*i_k_* | is the current amplitude. Representation of the current (real, imaginary, and modulus) versus frequency, at two different drain voltages. e,f) Complex plane representation of the currents. The arrows indicate the direction of increasing frequency.

The AC measurements of the currents *i_g_*, *i_d_* of the PEDOT:PSS OECT device in Figure 3 are shown in **Figure** **11** for two different drain voltages. Figure 11a,b,c,d displays the current as a function of frequency. We can appreciate some patterns in the response. For the gate current, there is a maximum of $i_{g}^{\prime }$ (blue) that marks a change of sign of 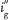 (orange) from positive to negative. This means a capacitive region followed by inductive effect according to the previous analysis. For the drain current, the $i_{d}^{\prime }$ begins with a region where it is constant and negative, and it turns to positive, and then shows a maximum. When $i_{d}^{\prime }$ increase, so does 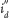, following the same trend, but the maximum of $i_{d}^{\prime }$ produces a change of sign of 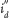, as in the case of *i_g_*. We also observe that higher *V_DS_* producer a higher $|i_{d}^{\prime }|$, as expected. Figure 11c, f shows the complex plane plot representation, with $i_{k}=i_{k}^{\prime }+\sqrt{-1}\;{i_{k}}^{\prime \prime }$. We can observe the transition from capacitive to inductive behavior in the *i_g_*. These properties wi104ll be well explained by the more general model suggested below.

**Figure 12:**
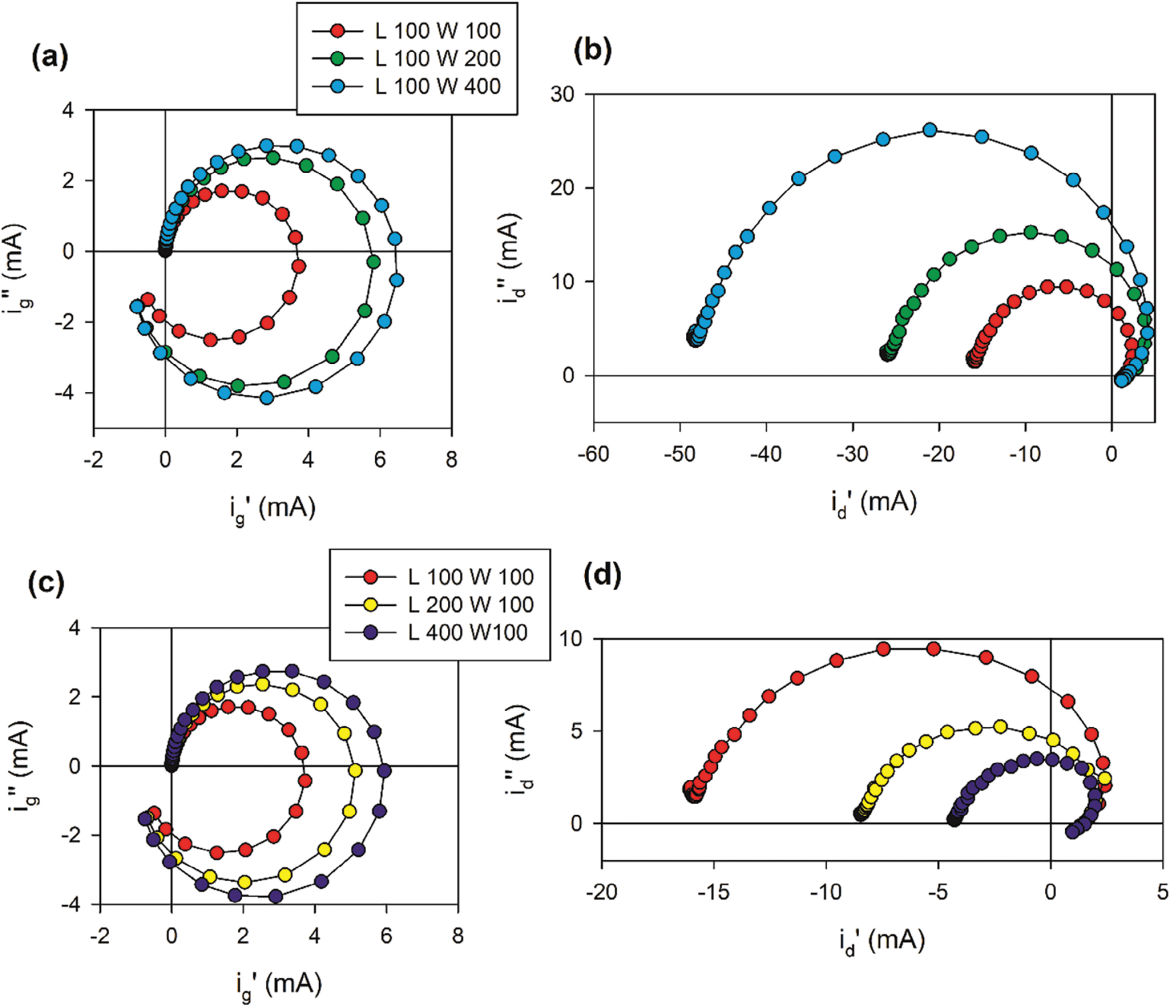
Experimental results for different transistors of channel dimensions *L* and *w* as indicated (in µm). *V_d_* =  100 *mV*, Complex plane representation of the gate (a,c) and drain (b,d) currents.

**Figure 13:**
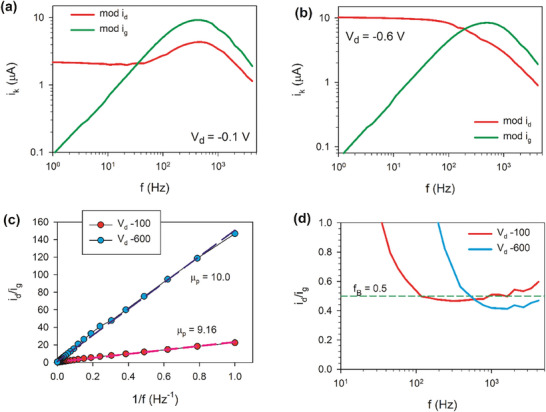
a,b) Experimental gate (|*i_g_* |) and drain (|*i_d_* |) current as a function of frequency for two different drain voltages, for a transistor of channel dimensions L = 800 µm, w = 100 µm, same data as Figure 10. c) Determination of the mobility (in cm^2^ V^−1^ s^−1^) from the current ratio method. (d) Determination of *f_B_* = *i_d_* /*i_g_*. The green line is a reference.

In **Figure** **12** we show the changes of admittances when the geometric dimensions of the transistor are modified. These properties will be interpreted later. In **Figure** **13** we show the experimental data related to the current ratio technique for the determination of mobilities at two different drain voltages. We can observe in Figure 13a,b a region of 90° phase lag between *i_d_* and *i_g_* at low frequency, and parallel lines at high frequency. Figure 13c shows the mobilities obtained by Equation (78). Figure 13d shows the determination of the parameter *f_B_* by Equation (79). More detailed interpretation will be presented below.

## 6 General Transmission Line Model

In this section, we formulate the model of the currents *I_g_* and *I_d_* in the transistor based on a general 2D transmission line approach developed recently.^[^ ^45^ ^]^ The goal of this Section is to express very precisely the foundations and limitations of the admittance models that are developed thereafter, as they are built on 1D simplified equations.

**Figure 14:**
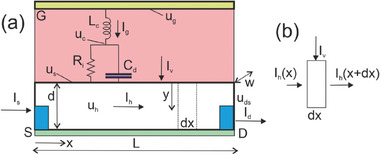
a) Scheme of the OECT. The pink zone is the electrolyte, yellow the gate contact, white the channel, and green the substrate of the film. *I_h_* is an electronic current, and *I_g_* is an ionic current. b) Local current conservation.

We consider an accumulation transistor in which holes of density *p* are balanced exactly by inserted anions of density *a*. The model characteristics, and the assignment of currents and potentials, are shown in **Figure** **14**a.^[^ ^45^ ^]^ As discussed earlier, *u_s_* is related to the gate voltage *u_g_* according to transport and polarization conditions in the electrolyte and its interfaces. An example consisting of ionic resistance *R_i_* in parallel with the double layer capacitance *C_d_* and a series inductor element *L_c_* is shown in Figure 14a.

The current conservation in the element of volume in Figure 14b is

(80) $$I_{h}\left(x\right)+I_{v}\;\left(x\right)=I_{h}\;\left(x+dx\right)$$

Hence, we obtain the balance of currents

(81) $$\frac{dI_{h}}{dx}=I_{v}\;$$

and

(82) $$\frac{dI_{h}}{dx}=-q\;w\;J_{v}\left(0\right)$$

To complete the model, it is necessary to state the equation for the vertical flux and how it fills the volume of the channel when the external voltage is modified. Integration of Equation (35) gives

(83) $$\frac{dA}{dt}=w\;J_{v}\left(0\right)$$

Therefore, the charging of a vertical slice in Figure 14b relates to the change of horizontal current as

(84) $$q\;\frac{dA}{dt}=-\frac{dI_{h}}{dx}$$

By Equation (81) we obtain Equation (21), that can also be expressed

(85) $$I_{v}=-q\frac{dA}{dt}$$

For a doped semiconductor, the formulas can be converted writing *A*  = *P*_0_  − *M*, where *P*_0_ is the intrinsic doping and *M* is the cation density, as described in Section 3.6.^[^ ^45^ ^]^ Correspondingly the sign of the derivatives must be changed as *dM*/*dt*  = − *dA*/*dt*, Equation (73).

We calculate in Equation (52) the derivative

(86) $$\frac{dI_{h}}{dx}=-\frac{qL}{\beta_{\mu }\tau_{e}}\frac{dA}{dx}$$

where

(87) $$\beta_{\mu }=\frac{1}{1+\frac{A}{\mu_{p}}\frac{d\mu_{p}}{dA}}\;$$

is a factor due to the density dependence of the mobility.^[^ ^88^ ^]^ Note that it can be β_μ_ < 0 if the mobility *d*μ_*p*_/*dA* < 0, which a well‐known property.^[^ ^88^, ^89^, ^90^ ^]^ In fact the decreasing mobility can be observed in Figure 3b.

The Equations (84) and (86) represent the transmission line approach, and they can be combined to

(88) $$\frac{dA}{dx}=\frac{\beta_{\mu }\tau_{e}}{L}\;\frac{dA}{dt}$$

The currents we seek, that can be measured from the contacts, are

(89) $$I_{d}=I_{h}\;\left(L\right)=-\frac{q\theta L}{\tau_{e}}\;A\left(L\right)$$

(90) $$I_{s}=I_{h}\;\left(0\right)=-\frac{q\theta L}{\tau_{e}}\;A\left(0\right)$$

By integration of (84) we find

(91) $$I_{h}\left(L\right)-I_{h}\;\left(0\right)=-q\int_{0}^{L}\frac{dA}{dt}dx$$

Now we define a *horizontal* average concentration

(92) $$A_{av}=\frac{1}{L}\;\int_{0}^{L}A\left(x\right)dx$$

Therefore

(93) $$I_{h}\left(L\right)-I_{h}\;\left(0\right)=-qL\frac{dA_{av}}{dt}$$

This is the representation of the general equilibrium of currents going in and out of the channel in a time‐dependent situation.

An integral of (88) has the result

(94) $$A\left(L\right)-A\left(0\right)=\theta \beta_{\mu }\tau_{e}\frac{dA_{av}}{dt}$$

Now we explain a method of solution^[^ ^44^ ^]^ to provide the separate currents (89, 90). We split (94) in two parts, using a fractional number *f_B_*:^[^ ^45^ ^]^

(95) $$A\;\left(L\right)=A_{av}+\theta f_{B}\beta_{\mu }\tau_{e}\frac{dA_{av}}{dt}$$

(96) $$A\;\left(0\right)=A_{av}+\theta \left(1-f_{B}\right)\beta_{\mu }\tau_{e}\frac{dA_{av}}{dt}$$

This procedure is similar to the partition of channel charge into drain and source parts.^[^ ^91^ ^]^ Hereafter we simply write the average concentration *A_av_* =  *A*. From (95) and (96) we get

(97) $$I_{d}=-\frac{q\theta L}{\tau_{e}}\;A-q\;L\;f_{B\;}\beta_{\mu }\;\frac{dA}{dt}$$

(98) $$I_{s}=-\frac{q\theta L}{\tau_{e}}\;A-q\;L\;\left(1-f_{B}\right)\;\beta_{\mu }\frac{dA}{dt}$$

Equation (97) was formulated in BM.^[^ ^44^ ^]^ Here it has been extended to include a mobility dependence on concentration by the modulation factor β_μ_.

The model of Figure 14 separates clearly two dominant capacitive effects: the double layer capacitance at the channel/electrolyte interface, and the chemical capacitance due to charging the ionic/electronic density of states of the film. The measurements can also reveal the geometric capacitance of the OMIEC, that often shows frequency dispersion due to dielectric relaxation phenomena.^[^ ^92^, ^93^, ^94^ ^]^

Let us establish the complete model to calculate the impedance (or in general, the response to any arbitrary stimulus). The vertical current *I_g_* is connected to the different voltages by Equation (21) and by the simplified diffusion Equation (45)

(99) $$I_{g}=-\frac{qL}{\tau_{d}}\left[A\left(u_{s}\right)-A\left(u_{h}\right)\right]$$

Considering the processes shown in Figure 12a at the electrolyte/channel interface, an ionic resistance *R_i_*, a double layer capacitance *C_d_*, and an inductor *L_c_* due to the contact wires, we have, in addition

(100)

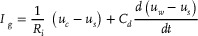

(101) $$u_{g}-\;u_{c}=L_{c}\;\frac{dI_{g}}{dt}$$

The horizontal current (94) can be written

(102) $$I_{d}=-q\frac{L}{\tau_{e}}\;A+L\;C_{tv}\frac{du_{h}}{dt}$$

where the transversal electronic capacitance is defined as

(103) $$C_{tv}=f_{B}\;\beta_{\mu }C_{\mu }$$

## 7 Impedance Model

### 7.1 Model with External Elements

To convert the previous Equations (21), (99)–(102) to linearized expressions we use the procedures explained in Section 3. We apply the Laplace transform, using *i_k_*, *v_k_* as the small signal current and voltage Laplace domain variables. For the equations involving the vertical current we obtain, in the electrolyte

(104) $$i_{g}=\left(\frac{1}{R_{i}}+C_{m}s\right)\;\left(v_{c}-v_{s}\right)$$

(105) $$i_{g}=\frac{v_{g}-v_{c}}{L_{c}s}\;$$

and inside the OMIEC layer

(106) $$i_{g}=\frac{1}{R_{d}}\;\left(v_{s}-v_{h}\right)$$

(107) $$i_{g}=Y_{\mu }\;v_{h}$$

where *R_d_* is the diffusion resistance and *Y*_μ_ is the admittance of the chemical capacitance, Equation (25).

The impedance in the electrolyte and its interfaces, *Z_s_*, is

(108) $$Z_{s}=\frac{1}{\frac{1}{R_{i}}+C_{d}s}+sL_{c}$$

We can write Equations (103), (104) and (104), (105) more generally:

(109) $$i_{g}=\frac{1}{Z_{s}}\;\left(v_{g}-v_{s}\right)$$

Equation (109) applies to any desired impedance *Z_s_* connected in series, according to the dominant interfacial capacitances and charge‐transfer or transport resistances.

We remark an important distinction that states whether the channel/electrolyte interface is allowing ion passage or not. Both cases occur in ionically‐controlled transistors.^[^ ^16^ ^]^ For an OECT, the ions enter the channel from the electrolyte, and it must be *Z_s_* (ω  =  0) = *R_i_* , i.e., a resistance. If, however, *Z_s_* (ω  =  0) =  ∞, the interface admits polarization but not ion insertion, as in an electrolyte‐gated field‐effect transistor^[^ ^95^, ^96^ ^]^ or in some perovskite transistors.^[^ ^36^, ^97^ ^]^

By (106, 107), the current inside the film, *i_g_* (*v_s_*, *v_h_*), is described by the simplified diffusion impedance *Z_d_* of Equation (39).

(110) $$i_{g}=\frac{1}{Z_{d}}\;v_{s}$$

**Figure 15:**
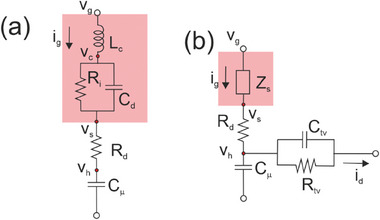
a) Equivalent circuit representation of the vertical impedance. b) Equivalent circuit representation of the transversal impedance.

These results complete the equivalent circuit of the vertical current of the transistor, that is indicated in **Figure** **15**a. *i_g_* charges the chemical capacitor, as stated in Equation (24). The *i_g_* also charges the diffusion impedance, Equation (110). Therefore, we have the relation

(111) $$v_{h}=\frac{1}{Y_{\mu }\;Z_{d}}\;v_{s}$$

The drain current *i_d_*, is obtained by linearization of Equation (56):

(112) $$i_{d}=\left(\frac{1}{R_{tv}}+C_{tv}s\right)\;v_{h}$$

The transversal electronic transport resistance is

(113) $$R_{tv}=\frac{\theta \;\beta_{\mu }\;\tau_{e}}{C_{\mu }}=\frac{\theta \;\beta_{\mu }L^{2}}{C_{\mu }\mu_{p}u_{ds}}\;$$

The electronic admittance *Y_el_* has the expression in Equation (65). In Figure 15b we draw the branch of horizontal transport that represents Equation (65). The effective relaxation time of the electronic channel is

(114) $$\tau_{h}=f_{B}\;\beta_{\mu }^{2}\tau_{e}$$

The total vertical impedance is defined as

(115) $$i_{g}=\frac{1}{Z_{v}}\;v_{g}$$

and a short calculation shows that

(116) $$Z_{v}=Z_{s}+Z_{d}$$

The electrolyte impedance and the diffusion impedance are connected in series by construction.

The vertical impedance is a conventional two‐electrode measurement, as remarked before in Figure 2a. Applying *v_g_* the vertical current *i_g_* charges the full impedance *Z_v_*. Comparing the relation between gate and internal film voltages is

(117) $$v_{h}=\frac{1}{Y_{\mu }\;Z_{v}}\;v_{g}$$

### 7.2 Model with Series Inductor

Now we choose a model that describes the components of the experimental data in Figures 11, 12, 13.

**Figure 16:**
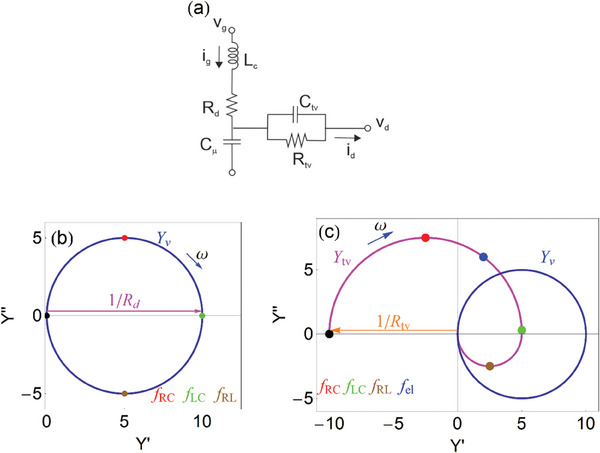
a) Equivalent circuit for the vertical admittance *Y_v_* and the transversal admittance *Y_tv_*. b,c) Complex plane plot representation of the admittances (in Ω^−1^ cm^−1^), indicating the characteristic frequencies and the direction of increasing frequency. The black point is at *Y*(ω  =  0). (b) admittance, c) transversal and vertical admittances. Parameters *R_d_* =  0.1 Ω cm, *C*_μ_ =  0.5 F cm^−1^, *Z_s_* =  *i*ω*L_c_*, *L_c_* =  5 × 10^−6^ H cm, *R_tv_* =   − 0.1 Ω cm,  *C_tv_* =  0.25 F cm^−1^.

The vertical admittance is defined in Equation (14) as *Y_v_* =  1/*Z_v_*. The vertical admittance is composed by the simplified diffusion admittance, Equation (39), and a series inductor, *Z_s_* =  *i*ω*L_c_*. It is represented in **Figure** **16**a and we have

(118) $$Y_{v}=\frac{1}{R_{d}}\;\frac{1}{1+\frac{\omega_{d}}{j\omega }+\frac{j\omega }{\omega_{RL}}}$$

In Figure 16b we observe the representation of the admittance *Y_v_* in the complex plot, which depicts a circle. As already mentioned, the vertical admittance is a conventional two‐contact model. The upper (positive) semicircle is due to the combination ω_*d*_ = *R_d_* *C*_μ_ and the lower (negative) semicircle is caused by the inductor‐resistance combination *R_d_L_c_*, with the frequency

(119)

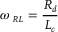




The maximum of the real part of admittance occurs at the characteristic frequency

(120)

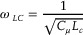

and the conductance at this point is 1/*R_d_*.

**Figure 17:**
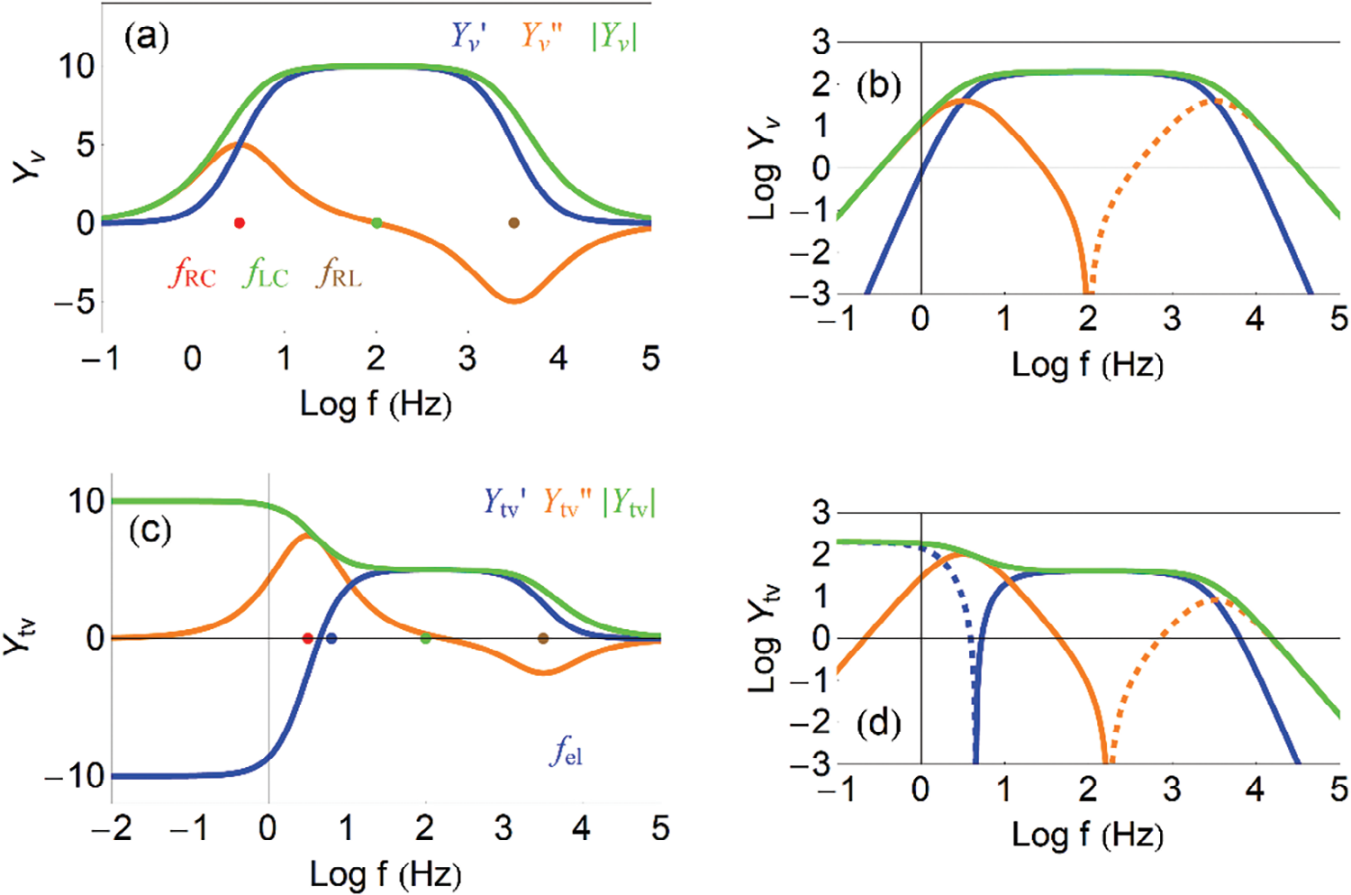
Vertical admittance *Y_v_* and the transversal admittance *Y_tv_* (in Ω^−1^ cm^−1^) for the model of Figure 16. Real and imaginary parts, and modulus of the admittance as a function of frequency. a,b) Vertical admittance in vertical axis linear (a) and log representation (b). c,d) Transversal admittance in vertical axis linear (c) and log representation (d).

The frequency dependence of the admittance is illustrated in **Figure** **17**a,b, showing the vertical axis both in linear (left) and log scale (right). The admittance 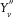 shows a positive capacitive peak at low frequency and a negative inductive peak at high frequency. The admittance turns down at frequencies higher than ω_*RL*_.

### 7.3 The Transversal Admittance

We complete the equivalent circuit model of Figure 16a by the analysis of the transversal admittance.

In Equation (68) we provide the transversal impedance in terms of the circuit components. Considering again that *Z_s_* =  *j*ω*L_c_*, *Y_v_* is given by Equation (118) and we have

(121) $$Y_{tv}=\frac{1}{R_{tv}}\;\frac{1+\frac{s}{\omega_{el}}}{1+\frac{s}{\omega_{d}}+\frac{s^{2}}{\omega_{LC}^{2}}}$$

We obtain the limits

(122) $$Y_{tv}\approx \frac{1}{R_{tv}}\;\omega \to 0$$

(123) $$Y_{tv}\approx \frac{C_{tv}}{C_{\mu }}\;\frac{1}{j\omega L_{c}}=f_{B}\beta_{\mu }\;\frac{1}{j\omega L_{c}}\;\omega \to \infty$$

The low frequency limit of the transversal admittance becomes constant, to the value of the transverse conductance. This can be observed in the complex plane representation of Figure 16c. Here *R_tv_* < 0 because θ  =   − 1 in Equation (55). This negative resistance does not have any meaning related to instability; it is just due to the convention of extracted current in the horizontal direction.^[^ ^45^ ^]^ The negative initial value of the admittance is also observed in the purple line of the complex plane representation, Figure 17c. The high frequency part is inductive, dominated by the vertical impedance. The difference in the two high frequency arcs of Figure 16c is an expression of the current ratio in Equation (79).

Figures 16 and 17 show that the equivalent circuit model of Figure 16a provides a very good representation of the main spectral features of the experimental AC currents shown in Figure 11.

### 7.4 Change of Dimensions

**Figure 18:**
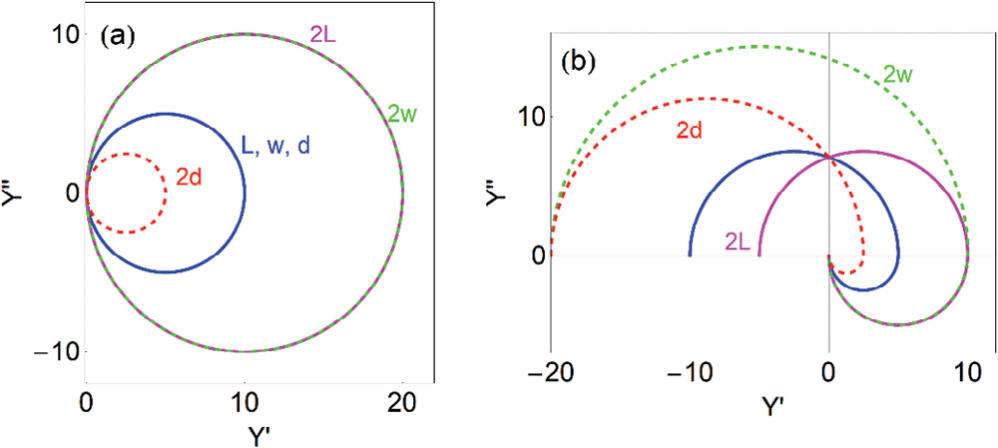
Complex plane representation of the equivalent circuit of Figure 16a for changing geometric dimensions. The blue line reference spectra correspond to Figure 16b. a) Vertical and b) transversal admittance.

For the analysis of the dependence of the admittances on the geometric dimensions, we summarize in **Table** **1** the relevant circuit elements derived in Section 3. The effect of changes in the various sizes is shown in **Figure** **18**.

**Table 1:** Summary of equivalent circuit elements.

Element	Expression
Chemical capacitance	*C*_μ_ = *Lwd* *q*|*da*/*du*|
Diffusion resistance	$\;R_{d}=\frac{d}{Lw}\;\frac{1}{3\;D_{ion}q\;\left|da/du\right|}$
Transversal resistance	$\;R_{tv}=\frac{L}{wd}\;\frac{\theta }{q\mu_{p}|V_{ds}\left|\;|da/du\right|}$
Transversal capacitance	*C_tv_* = *f_B_* *Lwd* *q*|*da*/*du*|

For increasing top area, the 1/*R_d_* increases, and for increasing thickness *d* it decreases, see in Figure 18a. In the transversal admittance, longer *L* increases *R_tv_*, and larger *w*, *d* decreases the transversal resistance, Figure 18b. These features explain well the variations observed in the experimental results of Figure 12.

### 7.5 Change of Drain Voltage

In Figure 16 we have provided a general model that explains well the main characteristics of the impedance and admittance of OECT. As a final check we go back to the two examples of Figure 11. They differ by the drain voltage, and this affects mainly the circuit element *R_tv_* indicated in Equation (122). In Figures 16 and 17 we provided simulations that describe the experimental trends of Figure 11, based on the insights about the structure of the admittance obtained in the previous sections.

**Figure 19:**
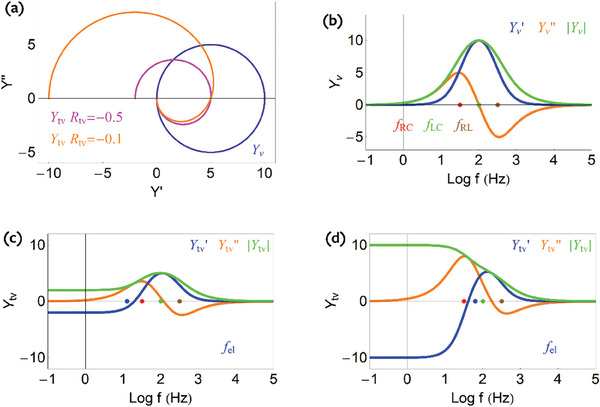
The vertical admittance *Y_v_* and the transversal admittance *Y_tv_* (in Ω^−1^ cm^−1^). a) Complex plane plot representation of the admittances. b–d) Real and imaginary parts, and modulus of the admittance, as function of frequency. b) Vertical admittance. c,d) Transversal admittance, at two different values of *R_tv_*, (c) − 0.5 Ω cm, d) − 0.1 Ω cm, representing different *V_d_* values. Parameters *R_d_* =  0.1 Ω cm, *C*_μ_ =  0.05 F cm^−1^, *Z_s_* =  *i*ω*L_c_*, *L_c_* =  5 × 10^−5^ H cm, *C_tv_* =  0.25 F cm^−1^.

We observe in Figure 11 that the measured *i_g_* are nearly the same for both drain voltages, as it should be expected, since the drain voltage does not directly affect the vertical impedance. A difference of the simulation in Figures 16 and 17 with respect to the experimental data in Figure 11, however, is that the intermediate plateaus of Figure 17, that corresponds to a resistance‐dominated region, are not observed in the data. Rather, the rising capacitive part in the *Y* − *f* plots is immediately continued with the declining inductive part. This is due to a closer value of ω_*d*_ to ω_*RL*_ in the experiment than in the simulations of Figure 17. Accordingly, in **Figure** **19** we modify the *C*_μ_ and *L_c_*, which brings the time constants together, see Figure 19b.

For a larger drain voltage, the *R_tv_* absolute value is expected to decrease, hence in Figure 11d there is a larger |*R_tv_* | value than in Figure 11b. The shift of *R_tv_* is also observed in the complex plane representation, Figure 11f. In Figure 19c,d, we change the *R_tv_* by a factor 5 and obtain a very good description of the spectral shape of the experimental data.

**Figure 20:**
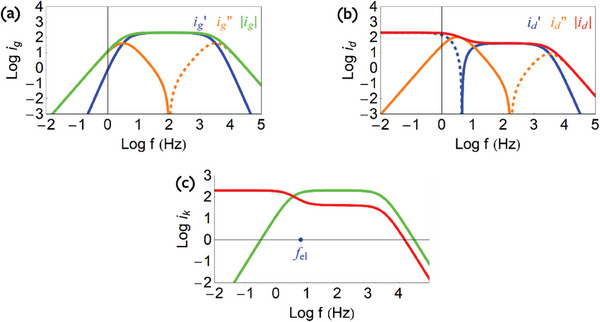
Simulation of frequency‐dependent (a) gate current (b) drain current and (c) their absolute values. *R_d_* =  0.1 Ω cm, *C*_μ_ =  0.5 F cm^−1^, *Z_s_* =  *i*ω*L_c_*, *L_c_* =  5 × 10^−6^ H cm, *R_tv_* =   − 0.1 Ω cm, *C_tv_* =  0.25 F cm^−1^.

The current ratio method was discussed in Section 4. In **Figure** **20** we show the different currents (a, b) and their ratio (c) for the model of Figure 19. The trends obtained are well satisfied by the experimental data of Figure 13.

### 7.6 The Horizontal Impedance

Another possibility of impedance measurement is the longitudinal impedance that can be obtained by modulating drain‐source voltage and measuring drain‐source current, as indicated in Figure 1b. This is a conventional two‐contact measurement that is often adopted to study carrier mobilities in organic layers.^[^ ^98^, ^99^ ^]^ Let us write Equation (96) as

(124) $$I_{d}=-\frac{q\mu_{p}A}{L}\;u_{ds}-q\;L\;f_{B\;}\beta_{\mu }\;\frac{dA}{dt}$$

By the small signal modulation of *u_ds_* we obtain

(125) $$i_{d}=-\frac{q\mu_{p}A}{L}\;v_{ds}$$

We can write also

(126) $$i_{d}=-\frac{1}{R_{ch}}\;v_{ds}$$

with respect to the channel resistance

(127) $$R_{ch}=\frac{L}{q\mu_{p}A}\;$$

To model the AC modulation of drain and source voltage in more detail it is necessary to go to the general transmission line equations and use the pertinent approximations. We have not attempted to derive such model so far. We anticipate that the horizontal transport pathway can couple with interfacial capacitances, and it may also induce diffusion currents, related to the horizontal charging.^[^ ^100^, ^101^, ^102^ ^]^

## 8 Conclusion

In summary, we described a new approach to the impedance measurements of ion‐controlled organic transistors, based on homogeneous concentrations, and incorporating the diffusion of ions inside the channel film. This paper developed a consistent model for the gate‐stimulated AC measurements that resulted in the vertical and transversal impedances. The model provided different spectral shapes that were closely validated by the experimental results. The vertical impedance can be analyzed by familiar methods, corresponding to intercalation impedance, dominated by the ion diffusion process, amply reported in the literature. The transversal admittance, however, shows in addition electronic components due to hole transport along the channel. At low frequencies the relation between the two impedances is purely capacitive, while at high frequency the admittances are related by the quotient of ionic and electronic capacitances. This result provides a new method to determine the contribution of the drain electrode to the transient current. The presented model is well‐suited to describe OECTs operating with small drain biases, where the drop in carrier concentration along the channel is nearly linear. In the future, the transversal admittance model could be extended to include contact resistance effects^[^ ^46^ ^]^ and by using a transmission line model for the channel to account for nonuniform carrier distributions.^[^ ^39^ ^]^

## Conflict of Interest

The authors declare no conflict of interest.

## Data Availability

The data that support the findings of this study are openly available in Zenodo at https://doi.org/10.5281/zenodo.13250692, reference number 13250692.
